# Median effective dose of spinal ropivacaine in combined spinal and epidural anesthesia for emergency cesarean delivery following failed vaginal delivery with epidural labor analgesia: a single-blind, sequential dose-finding study

**DOI:** 10.1007/s00540-024-03393-7

**Published:** 2024-08-28

**Authors:** Yu Wei, Shanshan Ye, Rui Ma, Tao Xu

**Affiliations:** 1 Department of Anesthesiology, the International Peace Maternity and Child Health Hospital, School of Medicine, Shanghai Jiao Tong University, Shanghai, China; 2 Shanghai Key Laboratory of Embryo Original Diseases, Shanghai, China; 3 Shanghai Municipal Key Clinical Specialty, Shanghai, China

**Keywords:** Ropivacaine, Combined spinal and epidural anesthesia, Median effective dose, Failed vaginal delivery

## Abstract

**Purpose:**

This study aimed to estimate the median effective dose of intrathecal isobaric ropivacaine without opioid required for adequate cesarean delivery anesthesia after epidural labor analgesia.

**Methods:**

Patients aged 20–40 years with American Society of Anesthesiology scores of I–II, body mass index ≤ 36, who underwent emergency cesarean delivery after failed vaginal delivery with epidural analgesia of a duration ≤ 6 h were included in the study. After removal of the epidural used for labor analgesia, a new combined spinal epidural was performed, and a dose of intrathecal isobaric ropivacaine without opioid was administered. The dose was determined using up–down methodology, with the starting patient's dose set to 12 mg. Adequate anesthesia, defined as a pinprick level no lower than T6 at 5 min after ropivacaine administration, resulted in the next patient receiving a dose of ropivacaine 1 mg higher, and inadequate anesthesia 1 mg lower. The primary outcome was the median (95% confidence interval (CI)) dose of spinal ropivacaine required for adequate cesarean delivery anesthesia.

**Results:**

Of the 46 patients included in the study, 40 were analyzed. The median spinal ropivacaine dose was 8.11 mg (95% CI 7.29–8.93 mg) by the Dixon and Mood method and 8.06 mg (95% CI 6.93–9.00 mg) by isotonic regression. Two patients had high spinal anesthesia.

**Conclusion:**

Our findings suggest that for 50% of patients undergoing cesarean delivery after failed vaginal delivery with epidural analgesia, an 8-mg spinal dose of isobaric ropivacaine without opioid provides an anesthesia level no lower than T6 at 5 min.

## Introduction

Continuous epidural analgesia is the optimal and most common method of analgesia for women undergoing labor in most hospitals. However, a transition to emergency cesarean delivery (EmCD) is required in 10–25% of patients [1–3]. In such situations, the options include general anesthesia, epidural top-up, combined spinal and epidural anesthesia (CSE), and spinal anesthesia (SA) alone, depending on maternal comorbidities, the urgency of the cesarean delivery, the availability of equipment, and the expertise of practitioners in obstetric anesthesia working in a particular country and treatment unit [4].

In China, women are not obliged to have a light or clear liquid diet during labor, and surgeons demand good muscle relaxation conditions. Such practices lead some anesthesiologists to prefer re-performing SA or CSE to general anesthesia and epidural top-ups when vaginal delivery is converted to cesarean delivery [5].

However, regular spinal doses of local anesthetics for elective cesarean delivery may lead to a high or total spinal block in patients undergoing EmCD after receiving epidural analgesia during labor [6]. This may result in maternal hypotension [5], threatening the safety of the mother and fetus [6]. Thus, our aim was to determine the median local analgesic dose of spinal ropivacaine. We hypothesized the spinal ropivacaine dose required in CSE for EmCD would be lower following epidural analgesia than that typically required for elective cesarean deliveries.

## Methods

The study was conducted in accordance with the Declaration of Helsinki and was approved by the Ethics Committee of the International Peace Maternity and Child Health Hospital (GKLW 2017–101). The study was registered at www.chictr.org.cn (ChiCTR1900027527) on November 17, 2019. Written informed consent was obtained from all the participants. This study adhered to the Consolidated Standards of Reporting Trials (CONSORT) guidelines.

Between December 2019 and June 2020, 46 women were recruited from the International Peace Maternity and Child Health Hospital. The inclusion/exclusion criteria were as follows:

### Inclusion criteria

(I) Women aged 20–40 years
(II) Patients with American Society of Anesthesiologists scores of I–II
(III) Full-term (> 37 gestational weeks), singleton pregnancies with a cephalic presentation
(IV) Satisfactory epidural analgesia defined as a numeric rating scale [NRS] score ≤ 3 during the contractions and a bilateral upper sensory level by pinprick above thoracic vertebra (T)10 when the patient was transferred to the operating room (OR).
(V) Patients undergoing EmCD following failed vaginal delivery with labor analgesia

### Exclusion criteria

(I) Contraindication to epidural or SA or accidental dural puncture during labor analgesia
(II) Coagulation disorders
(III) History of chronic pain
(IV) Obesity (body mass index [BMI] > 36 kg/m.^2^)
(V) Duration of epidural labor analgesia exceeding 6 h prior to EmCD
(VI) High analgesia levels by pinprick (higher than T6) prior to CSE
(VII) Previous spinal or dural puncture epidural analgesia for labor pain
(VIII) Patient refusal

### Epidural analgesia for labor pain

After entering the labor room, the researcher in charge of participant recruitment explained the study details, advantages, and disadvantages of re-performed CSE compared with epidural top-ups for the patients who requested epidural analgesia and met the first three inclusion criteria. Subsequently, the patients were administered Ringer’s lactate solution at a rate of 8 ml kg^−1^ h^−1^ after establishing an intravenous (IV) line by 16 G cannulation. After a cervical examination was performed by midwives and cervical dilatation beyond 3 cm was confirmed, an epidural puncture was conducted by an anesthesiologist at the anatomically determined L3–4 intervertebral interspace using a 17 G Tuohy needle. An epidural catheter was inserted in the cephalic direction and advanced 4–5 cm into the epidural space. After initiating the infusion with a 12-ml volume of solution containing 0.1% ropivacaine and sufentanil (0.4 μg ml^−1^), a patient-controlled epidural analgesia (PCEA) machine was connected to the patient. The PCEA fluid consisted of ropivacaine (200 mg), sufentanil (80 μg), and normal saline, with a total volume of 200 ml. The PCEA pump infused solution at 10 ml per hour, with a patient-initiated bolus of 6 ml and a lockout of 5 min.

### Anesthesia for cesarean delivery

After informing the patients of the advantages and disadvantages of re-performed CSE compared with epidural top-ups, those who met the inclusion criteria were recruited immediately after an obstetrician decided to transition to EmCD. Written informed consent was obtained before entering the OR. After entering the OR, routine monitoring was initiated, including electrocardiography, pulse oximetry, and non-invasive blood pressure monitoring. The heart rate, oxygen saturation, and blood pressure were recorded every 2 min. The anesthesiologist assessed the epidural analgesia level by pinprick in the bilateral midaxillary line. Infusion of Ringer’s lactate solution was maintained at a rate of 8 ml kg^−1^ h^−1^, and oxygen was administered at 3 L min^−1^. Subsequently, the patient was placed in the left lateral position, and the epidural catheter used for analgesia during labor was removed. An epidural puncture using a 17 G Touhy needle was conducted at the interspace of the L2–3 vertebrae (one upper interspace of the previous epidural catheter for labor analgesia) by the same anesthesiologist who performed the initial epidural puncture during labor analgesia. A 27 G Whitacre needle was inserted through the Touhy needle. After the cerebrospinal fluid was detected, the research dose of 0.5% ropivacaine (Naropin, AstraZeneca AB, Sodertalje, Sweden) was injected into the subarachnoid space by the anesthesiologist. Finally, a catheter was inserted in the cephalic direction and advanced 5 cm into the epidural space.

After the patient was moved to a supine position with left uterine displacement created by placing a wedge under the right hip, Ringer’s lactate solution was co-loaded and administered at a rate of 1 ml kg^−1^ min^−1^. If the anesthesia level assessed by pinprick in the anterior midline was below T6 5 min after spinal ropivacaine administration (evaluated by an observer), another bolus dose (5 ml) of 2% lidocaine was administered through the epidural catheter in 3 min intervals until the anesthesia level reached T6. The obstetrician began the operation once the anesthesia level reached T6. The observer recorded the NRS score and any complaints from the patient. Esketamine (15 mg) was administered intravenously if NRS scores were above 2 before delivery.

Hypotension was defined as a decrease of over 20% in systolic blood pressure relative to the baseline value. IV ephedrine (6 mg) was administered by the anesthesiologist every time a patient experienced hypotension. Bradycardia was defined as a heart rate of < 50 beats min^−1^; when bradycardia occurred, IV atropine (0.5 mg) was administered by the anesthesiologist.

Immediately after delivery, 1 ml of umbilical artery (UA) blood was collected by an obstetrician, and blood gas analysis was performed by a research assistant.

### Design of the sequential dose-finding study

This was a prospective, single-blind, up-and-down, sequential dose-finding study. The dose of spinal ropivacaine ranged from 6 to 12 mg. Based on a previous study [7], an initial dose of 12 mg ropivacaine was set as the ceiling dose and assigned to the first patient. The dose for the subsequent patient was increased or decreased by 1 mg depending on the response of the previous patient. If the anesthesia level assessed by pinprick was not below T6 at 5 min after spinal ropivacaine administration, the spinal dose of ropivacaine was considered a success. A lower spinal dose was then assigned to the next patient. Conversely, when the spinal dose of ropivacaine was considered a failure, a higher spinal dose was assigned to the next patient. All spinal doses of ropivacaine were prepared and injected into the subarachnoid space by the anesthesiologist to ensure that all patients were blinded to the ropivacaine dose used.

### Outcomes

The primary outcome was the spinal dose of ropivacaine required to ensure an anesthesia level no lower than T6 at 5 min for 50% of the patients, with its 95% confidence interval (95% CI). The secondary outcomes included maternal and neonatal observations. More specifically, maternal observations included the indications for EmCD, induction-to-delivery interval, duration of surgery, anesthesia level at the beginning of the cesarean section, the occurrence of a high-level block, rescue IV esketamine, administration of IV ephedrine for rescue during hypotension, the incidence of nausea and vomiting, maternal bradycardia, total IV fluids administered before delivery during EmCD, and epidural rescue bolus times required before surgery. Neonatal observations included the neonatal weight, Apgar scores 1 and 5 min after delivery, and pH and base excess values in the UA blood gas analysis. Other maternal characteristics were also recorded, such as age, weight, height, gestational week, gravidity, parity, duration of administration drug volume required for analgesia during labor, and sensory level before anesthesia.

### Sample size calculation

Simulation studies have suggested that including at least 20–40 patients can provide stable estimates of the target dose for most scenarios [8, 9]; thus, 40 patients were included.

### Statistical analysis

The Dixon and Mood method for up-and-down testing and isotonic regression analysis were used to estimate the median effective dose (ED_50_) of spinal ropivacaine and its 95% CI. These analyses were conducted using R software, version 3.4.4 (R Foundation of Statistical Computing, Vienna, Austria), with the R package “ed50” (V 0.1.1).

Data on characteristics and secondary outcomes were summarized as the mean ± standard deviation, median (interquartile range), median (range), or numbers and proportions, as appropriate, using Statistical Package for the Social Sciences (SPSS) software for Windows (version 24.0; SPSS Inc., Chicago, IL, USA).

## Results

**Fig. 1:**
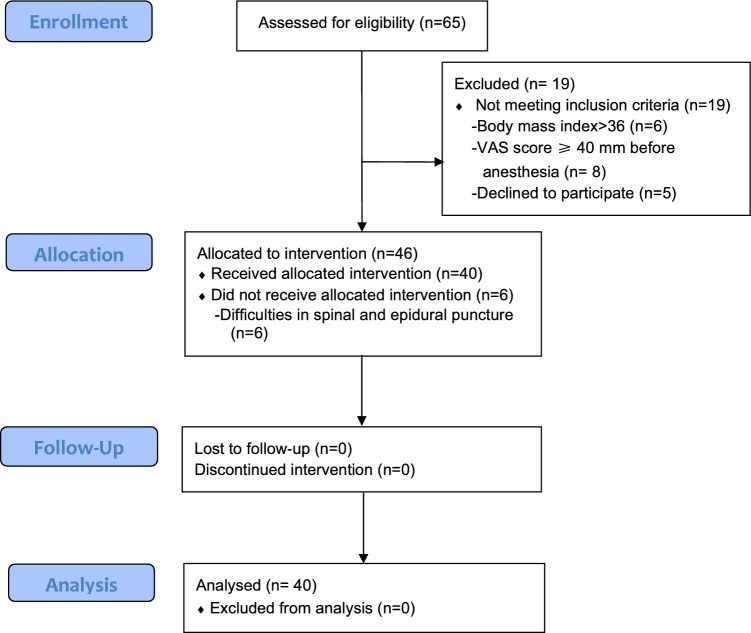
Diagram of the study

Between December 2019 and June 2020, 46 women were enrolled in the International Peace Maternity and Child Health Hospital. Six patients did not receive the allocated intervention owing to difficulties encountered while attempting the CSE puncture for EmCD; therefore, 40 patients were included in the final analysis (Fig. 1). Data related to the maternal demographic and epidural labor analgesia variables are shown in Table 1. Sixteen patients who failed to progress in the active phases of labor, five patients with meconium-stained amniotic fluid, and 19 patients with fetal distress were included in the study.

**Table 1:** Maternal demographic variables and situations of epidural labor analgesia

Characteristics	*n* = 40
Age, year	31.1 ± 3.7
Height, cm	161.5 ± 4.1
Weight, kg	68.7 ± 6.5
Gestational period, weeks	39.0 ± 1.1
Body mass index	26.3 ± 2.2
Gravidity, n	2(1–2)^a^
Parity, n	0(0–0)
Labor analgesia duration, min	237.3 ± 72.4
Drug volume for labor analgesia, mL	30.3 ± 6.4
Analgesia level to pinprick	T9(T8-T10)^a^

Data are presented as mean ± standard deviation

^a^median (interquartile range) as appropriate

**Fig. 2:**
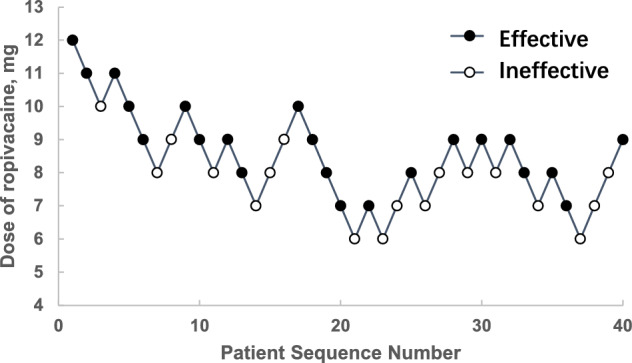
The patient allocation sequence and the response to the assigned spinal ropivacaine dose for EmCD after failed vaginal delivery by epidural labor analgesia. The patient sequence number (X-axis) is the order of patient exposures by up-and-down sequential design. The assigned dose level are present on Y-axis. An effective dose is denoted by a solid circle, while an ineffective one is denoted by a hollow circle. EmCD, emergency cesarean delivery

Figure 2 shows the sequence of the effective and ineffective responses to each dose of spinal ropivacaine in the 40 successive patients. Ropivacaine doses ranging from 6 to 12 mg were used. The ED_50_ of spinal ropivacaine was determined to be 8.11 mg (95% CI 7.29 to 8.93 mg) or 8.06 mg (95% CI 6.93 to 9.00 mg) based on the Dixon and Mood method or isotonic regression analysis, respectively, among the patients undergoing EmCD following failed vaginal delivery with epidural labor analgesia.

Data for the maternal outcome variables are presented in Table 2. The mean time interval from induction to delivery was 16.1 ± 4.3 min, and the duration of surgery was 46.3 ± 8.0 min. The sensory level was determined to be around T6 (T4–T6) at the beginning of the cesarean section, and no patient needed IV esketamine before delivery. Of the 40 patients, the numbers and percentages who experienced side effects were as follows: two patients (5%) exhibited a high-level block (upper sensory level above T4), six (15%) experienced hypotension requiring administration of IV ephedrine for rescue, seven (17.5%) experienced nausea and vomiting, and two (5%) experienced bradycardia. The total co-loaded IV fluid volume of Ringer’s lactate solution was 350 (300–400) ml.

**Table 2:** Maternal outcomes

Outcomes	*n* = 40
Indications for EmCD (Failure to progress/Meconium-stained Amniotic fluid/Fetal distress)	16/5/19
Induction to delivery interval, min	16.1 ± 4.3
Duration of surgery, min	46.3 ± 8.0
Anesthesia level at the beginning of EmCD	T6(T4-T6)^a^
High-level block (upper anesthesia level above T4), *n* (%)	2 (5%)
IV esketamine, *n* (%)	0 (0%)
IV rescue ephedrine, *n* (%)	6 (15%)
Nausea and vomiting, *n* (%)	7 (17.5%)
Maternal bradycardia, *n* (%)	2 (5%)
Total IV fluid before delivery during EmCD, mL	350(300–400)^b^

Data are presented as mean ± standard deviation

*EmCD* emergency cesarean delivery, *IV* intravenous

^a^median (interquartile range), ^b^median (range) and number (percentage) as appropriate

Table 3 shows the epidural rescue boluses before surgery in each subgroup. In the 11 and 12 mg spinal ropivacaine subgroup, no extra epidural rescue bolus was needed to achieve a successful anesthesia effect. In the 9, 10 mg spinal ropivacaine subgroup, 20% or 25% of patients needed one epidural rescue bolus, respectively, and no patients needed two rescue boluses. In the 7, 8 mg spinal ropivacaine subgroup, 25% or 41.7% of patients needed one epidural rescue bolus, and 37.5% or 16.6% needed two epidural rescue boluses, respectively, to achieve a successful anesthesia effect. In the 6 mg spinal ropivacaine group, 100% of patients required two epidural rescue boluses.

**Table 3:** Epidural rescue boluses before surgery in each subgroup

	Spinal ropivacaine dose (mg)						
	6 (*n*=3)	7 (*n*=8)	8 (*n*=12)	9 (*n*=10)	10 (*n*=4)	11 (*n*=2)	12 (*n*=1)
Epidural rescue boluses							
0	0 (0)	3 (37.5)	5 (41.7)	8 (80.0)	3 (75.0)	2 (100.0)	1 (100.0)
1 (5 mL)	0 (0)	2 (25.0)	5 (41.7)	2 (20.0)	1 (25.0)	0 (0)	0 (0)
2 (10 mL)	3 (100.0)	3 (37.5)	2 (16.6)	0 (0)	0 (0)	0 (0)	0 (0)

Data are presented as number (percentage)

Data on neonatal outcomes are presented in Table 4. The mean weight of the 40 neonates was 3,201 ± 484 g. The Apgar scores of the neonates at 1 min and 5 min were 10 (10–10) and 10 (10–10), respectively, and no neonate exhibited an Apgar score < 7 at 1 min or 5 min. The pH and base excess values of the UA samples were 7.319 ± 0.053 and − 3.76 ± 2.15 mEq l^−1^, respectively.

**Table 4:** Neonatal outcomes

Outcomes	*n* = 40
Neonatal weight, g	3201 ± 484
Apgar scores at 1 min	10(10–10)^a^
Apgar scores at 5 min	10(10–10)^a^
pH of UA	7.319 ± 0.053
BE of UA, mEq/L	− 3.76 ± 2.15

Data are presented as mean ± standard deviation

*UA* umbilical artery, *BE* base excess

^a^median (interquartile range) as appropriate

## Discussion

Among patients undergoing EmCD after failed vaginal delivery with epidural labor analgesia, the ED_50_ of spinal ropivacaine without opioid was determined to be 8.11 mg (95% CI 7.29 to 8.93 mg) based on the Dixon and Mood method and 8.06 mg (95% CI 6.93 to 9.00 mg) based on isotonic regression analysis.

CSE, SA, epidural top-ups, and general anesthesia can all be used in EmCD. Anesthesiologists may select a mode of anesthesia based on maternal comorbidities, urgency of cesarean delivery, availability of equipment, and expertise of practitioners of obstetric anesthesia in a particular country and treatment unit [4].

General anesthesia is usually used as a last resort when spinal or epidural anesthesia has failed [4, 10] or in urgent situations [11]. It is associated with a high risk of failed tracheal intubation [4, 10, 12], and a 5-min Apgar score < 7 [11, 13]. The unrestricted intake of food and drink strategy during labor in China further increases the risks of reflux and aspiration during general anesthesia. Epidural top-ups after labor analgesia have the advantage of avoiding the need for a second puncture and shortcomings of a relatively longer induction-to-delivery interval [11] and inadequate anesthesia [14].

CSE and SA have the advantages of shorter duration of action [14] and better perioperative or postoperative analgesia [15]. They are also safer for obstetric patients with full stomachs or difficult airways. These desirable outcomes have led many obstetric anesthesiologists to prefer CSE and SA in EmCD cases [5, 15, 16]. High-level blocks or hypotension has been reported following CSE or SA [5, 6, 16, 17], which may lead to adverse maternal and fetal outcomes [5, 16] and can decrease the degree of satisfaction with the procedure. In the case of EmCD after failed vaginal delivery with epidural labor analgesia, the incidence of high-level blocks and hypotension may be higher if a regular spinal dose for elective cesarean delivery is used [17]. Leakage of local anesthetic from the epidural to the subarachnoid space [18] and the reduction in the volume of subarachnoid space by previous epidural labor analgesia fluid [17] have been implicated as causes of higher spinal blocks. Thus, a spinal dose that adequately balances the safety and efficacy of anesthesia should be determined for EmCD after failed vaginal delivery with epidural labor analgesia.

Ateser et al. [19] reported a 15 mg spinal regimen of isobaric ropivacaine could achieve satisfactory effect for elective cesarean, and Oraon et al. [20] proved a 12 mg of intrathecal isobaric ropivacaine provided adequate anesthesia with lower incidence of hypotension for elective cesarean. Both were much higher than the estimated ED_50_ of spinal isobaric ropivacaine in CSE for EmCD in current study, which proved our hypothesis of a reduction of spinal ropivacaine dose after failed vaginal delivery with epidural labor analgesia.

Notably, the failure rate of CSE punctures in this study was relatively higher than that in elective cesarean deliveries [21]. We deduced that the fuzzy sensation of breaking through the dura and the expansion of the space between the dura mater and ligamentum flavum due to prolonged immersion of epidural fluid after labor analgesia led to puncture failure. Thus, women with epidural labor analgesia duration exceeding 6 h prior to EmCD were excluded from the study. To decrease the time spent before EmCD while avoiding the risks of general anesthesia, rapid epidural catheterization after a maximum of two attempts of spinal puncture in 3 min, followed by epidural anesthesia through a catheter is suggested.

The current study has several limitations. First, even if the epidural catheter used for labor analgesia was functional when an emergency cesarean was needed, the catheter was removed, and a combined spinal epidural anesthesia was performed, which may have led to additional trauma to the patient and caused some ethical problems. However, all women were informed about the disadvantages of re-puncture before recruitment, the possibility of a better anesthetic effect of a re-puncture, and the earlier commencement of surgery. Most were willing to participate in the study. Second, the patients included needed to have a BMI of ≤ 36 kg/m^2^ and an analgesia time of ≤ 6 h, which limits the generalizability of the results. Further research should be performed to evaluate effectiveness and safety in patients with higher BMIs or longer analgesia time. Third, the use of fentanyl or sufentanil as an adjuvant may further reduce the ED_50_ of spinal ropivacaine required for analgesia and the occurrence of adverse effects in patients undergoing EmCD with CSE or SA [22]. However, those drugs were not used in the present study because a single drug may reduce the risk of error in an emergency. Further studies are needed to estimate the ED_50_ of spinal ropivacaine in combination with fentanyl or sufentanil.

The current study suggests that for 50% of patients undergoing cesarean delivery after failed vaginal delivery with epidural analgesia, an 8-mg spinal dose of isobaric ropivacaine without opioid provides an anesthesia level no lower than T6 at 5 min.

## Data Availability

The dataset used and analyzed during the current study available from the corresponding author on reasonable request.
